# Transcytosis of TrkA leads to diversification of dendritic signaling endosomes

**DOI:** 10.1038/s41598-018-23036-8

**Published:** 2018-03-16

**Authors:** Kelly Barford, Austin Keeler, Lloyd McMahon, Kathryn McDaniel, Chan Choo Yap, Christopher D. Deppmann, Bettina Winckler

**Affiliations:** 1 Department of Cell Biology, University of Virginia, 1340 Jefferson Park Avenue, Charlottesville, Virginia 22908 USA; 2 Department of Biology, University of Virginia, Physical Life Sciences Building (PLSB), 90 Geldard Drive, Charlottesville, Virginia 22903 USA; 3 Department of Biomedical Engineering, University of Virginia, Charlottesville, Virginia 22903 USA

## Abstract

The development of the peripheral nervous system relies on long-distance signaling from target organs back to the soma. In sympathetic neurons, this long-distance signaling is mediated by target derived Nerve Growth Factor (NGF) interacting with its axonal receptor, TrkA. This ligand receptor complex internalizes into what is commonly referred to as the signaling endosome which is transported retrogradely to the soma and dendrites to mediate survival signaling and synapse formation, respectively. The molecular identity of signaling endosomes in dendrites has not yet been determined. Here, we perform a detailed analysis of TrkA endosomal compartments and trafficking patterns. We find that signaling endosomes are not uniform but molecularly diversified into Rab7 (late endosome) and Rab11 (recycling endosome) populations in axons and dendrites *in vitro* and in the soma *in vivo*. Surprisingly, TrkA-NGF signaling endosomes in dendrites undergo dynamic trafficking events, including putative fusion and fission. Overall, we find that signaling endosomes do not remain as a singular endosomal subtype but instead exist in multiple populations that undergo dynamic endosomal trafficking events. These dynamic events might drive functional diversification of the signaling endosome.

## Introduction

The development of the sympathetic and sensory nervous systems requires the target derived neurotrophic factor, Nerve Growth Factor (NGF)^1^. The molecular and cellular mechanisms that enable target derived NGF to transmit signals long distances from distal axons to the cell body have been under intense investigation for several years. NGF binds to its receptor TrkA in axon terminals, and the NGF-TrkA complex is internalized into what is commonly referred to as the signaling endosome (SE) because of its capacity to maintain active signaling cascades such as MAPK, PI3K, and PLC-gamma^2–5^. The SE engages retrograde axonal transport machinery to traffic back to the cell body using the minus-end directed microtubule motor dynein^6^. Once it reaches the soma, the SE is able to maintain signaling cascades for over 6 hours^7^. These signaling pathways include activation of pro-survival transcription factors like CREB^8^ and suppression of apoptotic machinery like Bax^9^. There is mounting evidence that the SE mediates much more than just survival, including dendrite development, synapse formation, changes in metabolism, and acquisition of neurotransmitter phenotypes^10–13^. Ginty and colleagues previously showed that SEs are not confined to the soma, but can travel to dendrites and signal for postsynaptic clustering^10^. Dendritic SEs reside near nascent post-synaptic specializations and mediate post-synaptic density (PSD) clustering both *in vitro* and *in vivo*^10,11^. Indeed, axonally-derived TrkA SEs continue to signal in the dendrite, and inhibition of TrkA phosphorylation in the dendrite abolishes PSD clustering^11^. In addition, we showed previously that SEs arriving in the soma can undergo exocytosis, and this pool of axonally-derived TrkA can subsequently re-endocytose^7^. We refer to this trafficking pathway as “retrograde transcytosis”, and we refer to the axonally-derived TrkA endosomes that undergo retrograde transcytosis as “post-trancytotic signaling endosomes” (PTSEs, Supplementary Figure 1D–E). This nomenclature is used to distinguish naïve TrkA undergoing anterograde transcytosis to the axon^14^ from the pathway we are investigating in this study (Supplementary Figure 1E). Currently, nothing is known about PTSEs, including whether they are motile, whether they are found in dendrites, and what their endosomal identity is.

Much is known about endosomal pathways in other cell types, especially fibroblasts, and for other internalized receptors, such as transferrin receptor. From these non-neuronal model systems, it is well established that the fate of endocytosed cargos is regulated by several endosomal members of the Rab family of small GTPases, especially Rab5 (early endosome), Rab7 (late endosome), and Rab11 (recycling endosome)^15^. Endocytosed cargos first arrive at the Rab5-positive early endosome (also marked by Early Endosomal Antigen 1, EEA1), from where they can sort into various arms of the endosomal pathway. From the EEA1+ early endosome, cargo can undergo distinct fates when sorted into segregated domains (reviewed in^16^). These domains are then released through fission to create individual endosomes^16^. For the purpose of this work, we will focus on sorting into the recycling and degradative arms of the endosomal pathway. Recycling-competent cargos are removed from the early endosome by sorting and fission events to generate endosomes positive for Rab11. In contrast, degradative cargos are typically sorted into Rab7-positive late endosomes which ultimately fuse with lysosomes. The TrkA SE also seems to follow these maturational rules. TrkA endocytosed at the distal tips of axons initially sorts into the early endosome^2^. From there, TrkA signaling endosomes have been identified to travel retrogradely back to the soma in the early endosome, or in a late endosome for transit^2,17^.

We used a Flag-TrkA knockin mouse and a Flag antibody feeding assay in combination with sympathetic neurons cultured in microfluidic chambers to ask how SEs mature^7,10^. We find that SEs exist in multiple endosomal populations beginning in the axon. Axonal SEs exist in equal populations of Rab11 (recycling) and Rab7 (late) endosomal compartments. Interestingly, dendritic SEs are frequently post-transcytotic and are also found in Rab11 (recycling) and Rab7 (late endosomal) pathways in roughly equal proportions. Live imaging of SEs in dendrites reveals frequent dynamic events, including presumptive fissions and fusions. Surprisingly, we also observe high levels of co-localization of re-endocytosed retrograde TrkA with simultaneously endocytosed transferrin, arguing that SEs do not sort into distinct pools of Rab11 endosomes, but mix extensively with other endocytosed cargos. In addition, we show that this endosomal diversification exists *in vivo*. We propose that two pathways contribute to SE diversification. First, TrkA in axons is found in both Rab11- and Rab7-positive endosomes. Second, axonally-derived TrkA can undergo retrograde transcytosis, and then sort equally into recycling (Rab11) and pre-degradative (Rab7) pathways. We propose that transcytotic resorting of internalized axonally-derived TrkA from early endosomes into Rab11- and Rab7-positive endosomal subpopulations results in SE molecular diversification.

## Materials and Methods

### Animals

All animal use was approved by the University of Virginia IACUC guidelines by protocol #3422 for the Winckler lab, and protocol #3795 for the Deppmann lab. All methods and experiments were performed in accordance within these guidelines and regulations. All animals were maintained in a C57BL/6 background. Both sexes were used. *TrkA*^*FLAG/FLAG*^ animals were a gift from D. Ginty, and genotyping was performed as previously described^10^.

### Compartmentalized Sympathetic Neuronal Cultures

Sympathetic neurons were dissected from the superior cervical ganglia of P1 mice as previously described. Dissociated neurons were plated in microfluidic devices as previously described^18^ and experiments were performed on DIV 10–15. Neurons were maintained in DMEM +10% FBS, penicillin/streptomycin, gentamycin, and 80–100 ng/mL NGF. For the first 5 days of culture, neurons were additionally maintained with 5 μM Ara-C to kill proliferating glia.

### Antibody feeding assays

For fixed cells, anti-FLAG antibody feeding experiments were performed as previously described^7^. Briefly, neurons were deprived of NGF overnight in the presence of a broad-pan caspase inhibitor BAF and anti-NGF antibody (Millipore Cat# AB1528SP, RRID:AB_90742) (1 µg/mL anti-NGF, 1 µg/mL BAF). Neurons were washed with DMEM + 10% FBS, and NGF (100 ng/mL) and anti-FLAG antibody (M1, Sigma-Aldrich Cat# F3040, RRID:AB_439712) were applied exclusively to the distal axon chamber for 30 minutes. The distal axon chamber was then acid washed with pH 2.0 for 30 seconds to remove remaining surface-bound antibody followed by PBS and media washes. An Alexa-647 anti-mouse antibody (Life technologies A-31571, RRID: AB_2536181, 1:400) was applied to the cell body chamber after washes to label post-transcytotic signaling endosomes (PTSEs).

For transferrin-feed experiments, neurons were changed to DMEM with no FBS for 1 hour prior to the experiment. The anti-FLAG antibody M1 feeding was performed as above. In addition to the Alexa-647 anti-mouse antibody, a Cy3 Transferrin (Jackson ImmunoResearch Labs Cat# 015–160–050, RRID:AB_2337214) was added at a concentration of 40 ng/mL for 2 hours.

For live imaging, neurons were deprived of NGF overnight in the presence of a broad caspase inhibitor as described above. Neurons were washed with normal media, and NGF (100 ng/mL) was applied to the distal axon chamber. To identify retrograde SEs, a Cy3-coupled anti-FLAG antibody (M2-Cy3, Sigma-Aldrich Cat# A9594, RRID:AB_439700) was applied with NGF to the distal chamber. Neurons were washed with PBS after 30 minutes and imaged at the indicated times. For PTSE labelling, non-labelled anti-FLAG antibody (M1, Sigma) was added to the distal axon chamber with NGF as described above and washed off after 30 minutes. An Alexa-568 anti-mouse antibody (Life Technologies A10042, RRID: AB_2534017, 1:100) was then added to the cell body compartment for 4–6 hour, washed with DMEM +10% FBS and imaged.

### Immunocytochemistry

Cells were fixed with 4% PFA for 15 min at room temperature. Cells were permeabilized and blocked with 1% BSA, 0.2% Triton X-100 for 10 minutes at room temperature. Primary antibodies were diluted in 1% BSA and applied overnight at 4 °C. Secondary antibodies were diluted in 1% BSA and applied at room temperature for 1 hour. For Lambda Phosphatase treatment, after fixation and permeabilization, neurons were incubated with Lambda Phosphatase solution overnight at 4 °C. Lambda Phosphatase solution:1 X PMP buffer (New England Biolabs, Cat# B0716), 1 X Mn^+^ buffer (New England Biolabs, Cat# B1761S), 2.4:100 dilution of Lambda Phosphatase (Santa Cruz, sc-200312). Neurons were washed and antibodies applied as described above.

### Antibodies

Rab7 antibody (Cell Signaling Technology Cat# 2094, RRID:AB_2300652) was used at a dilution of 1:200 in 10% BSA. This antibody has been validated after siRNA-mediated knockdown on western blots^19^. It has additionally been validated after knockdown and immunofluorescent staining^20^. Validation after knockdown was also carried out in our lab for neuronal cultures (data not shown). Rab11 antibody (Cell signaling technology, Cat# 3539 S, RRID: AB_2253210) was used at a dilution of 1:200. This antibody was validated by siRNA knockdown and western blot^21^. It has additionally been validated through knockdown and immunofluorescent staining^22^. EEA1 antibody (Cell Signaling Technology Cat# 2411 S, RRID:AB_2096814), was used at a dilution of 1:200. This antibody staining is consistent with early endosomes, co-localizing with Transferrin, but not Rab7^23^. In our hands, this antibody recognized overexpressed tagged EEA1^24^, co-localizes with endocytosed Tfn at short but not long chase times, the staining does not co-localize with degradative cargos, and the staining disappears when early endosomes are disrupted by Rab5 interference^25^.

### Rab Co-Localization

**Figure 1:**
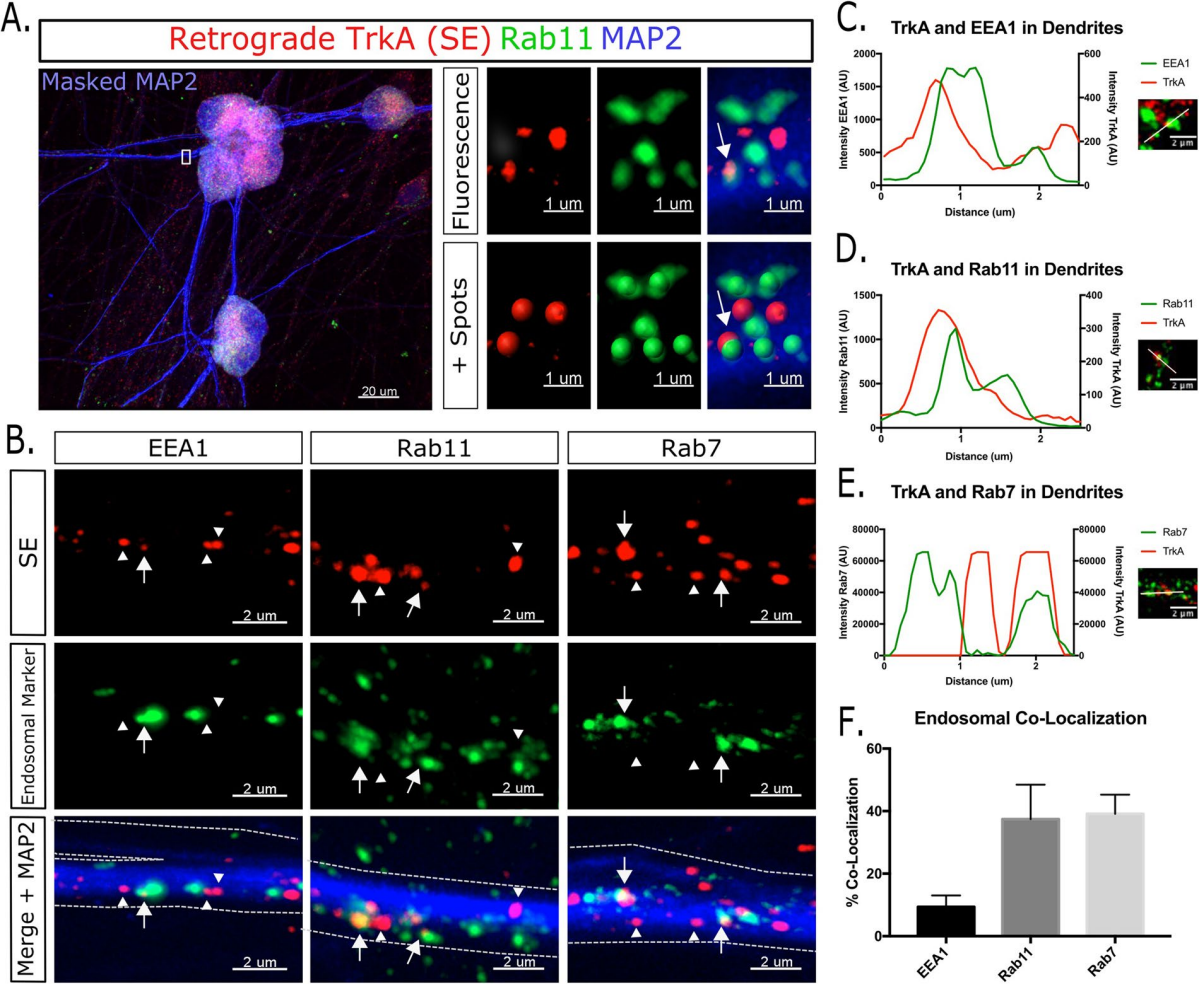
Dendritic SEs exist in multiple different endosomal populations. (**A**) Illustration of Imaris spot co-localization. The endosome on the bottom left of the inset is co-localized (arrow), while the other two SEs are not. (**B**) SEs were fixed after 2–6 hours of distal anti-FLAG antibody feeding and co-stained for endosomal markers EEA1, Rab7, and Rab11 as indicated. Arrows are co-localized, arrowheads are not. Individual channels and merged images shown. Dendrites are outlined with dotted lines. (**C**–**E**) Representative line scans with corresponding images showing intensity peaks of EEA1 (**C**), Rab11 (**D**), and Rab7 (**E**) in green and TrkA SEs in red. Co-extensive peaks indicate co-localization. (**F**) Quantification of dendritic SE co-localization with endosomal marker (n = 3–4 independent experiments, 15 fields of view per experiment).

Images of fixed cells were acquired on a Zeiss AxioZoom Oberserver.Z1 with Apotome 2.1 structured illumination and acquired with a 63x oil objective as Z-stacks. Imaris software was used for quantification. A 3 dimensional mask of the dendrites was made from the MAP2 channel. From the mask, each individual channel was isolated within the mask. Masked images were blinded, and thresholds for signal were set against a t = 30 minute time point, when there are very few retrograde signaling endosomes in dendrites. The Imaris spot function was used to identify endosomes within the MAP2 masked volume. Endosomes were considered co-localized if the intensity centers were within 0–0.4 µm apart (Fig. 1A). This was chosen based on the pixel size of ~250 nm × 250 nm × 450 nm in the x-y-z dimensions in our imaging system, therefore all co-localization is within one voxel. All quantification is presented as the average ± standard deviation.

### Movie Analysis

Movies were taken on a Zeiss-880 confocal microscope at a rate of 1 frame/sec. Instantaneous speeds were determined with the software Kymograph Clear and Kymograph Direct as previously described^26^. Kymographs are a two-dimensional display of a movie whereby a line scan of each movie frame is sequentially assembled to show behavior of endosomes over time. The x-axis represents space along the process, with the cell body being towards distance “0”, whereas the y-axis represent time. All quantification is presented as the average ± standard deviation. To determine directionality, color-coded kymographs from KymoClear (blue = stationary, red = anterograde, green = retrograde), were used. Line scans were drawn at 3 locations per kymograph and the number of peaks associated with retrograde, anterograde, and stationary events were quantified.

### Animal Injections

Postnatal day 21 ± 2 days mice were initially anesthetized in a chamber with 4% isofluorane and subsequently maintained under anesthesia at 2% isofluorane with a hood. Eyes were coated in paralube and the fur covering the throat and upper chest removed by Nair. After thorough washing with water, the area was amply coated in betadine and then washed with isopropanol. The right salivary gland was palpated out and held in place while 2 µl of material PBS or M2-Cy3, were injected in slowly over 30 seconds using a 30 and 1/2 gauge needle and a Hamilton syringe. The needle was held in place an additional 15 seconds to prevent backflow. The animals were given 25 mg/kg ketoprofen as a post-injection analgesic. The animals were maintained for 4–5 hours before euthanasia and the dissection of both SCGs.

### Immunohistochemistry

SCGs of salivary gland injected animals were flash-frozen in 2-methylbutane. Cryosections of SCGs were cut at 20 um thickness. Sections were warmed to RT for 30 minutes and submerged in 4% PFA for 15 minutes at RT. Sections were then subject to antigen retrieval and antibody staining as previously described^27^.

## Results

### Multiple pools of signaling endosomes exist in the dendrites

Dendritic SEs were previously shown to carry out important signaling functions with respect to postsynaptic maturation^11^. Additionally, at least a subset of the SEs that enter the dendrites have been shown to be actively signaling, as marked by the presence of phosphorylated TrkA^11^. However, their molecular composition is not known. To investigate this, we took advantage of sympathetic neurons isolated from FLAG-TrkA knock-in mice, *TrkA*^*FLAG/FLAG*^, which have a FLAG tag knocked into the endogenous TrkA locus in frame with the extracellular domain of the protein^10^. These neurons were grown in microfluidic devices which allows for fluidic separation of axons and somas (Supplementary Figure 1A–B). SEs, decorated by feeding anti-FLAG antibody from the axon chamber, reach the soma starting at about 30 minutes after feeding and continue to accumulate for several hours. This system thus allows for assaying NGF-dependent transport of TrkA-SEs to the soma for signaling. To test specificity of the anti-FLAG antibody and integrity of the chambers, neurons from either *TrkA*^*FLAG/FLAG*^ mice or WT mice (not containing FLAG-TrkA) were incubated with the NGF/anti-FLAG antibody at 37 °C for 30 minutes. As previously described, no staining was observed in WT cultures, demonstrating that the antibody does not enter axons non-specifically (Supplementary Figure 1C) and can thus be used to faithfully track NGF-TrkA SEs. Additionally, we have developed advanced quantification techniques to analyze endosomal co-localization. Using Imaris software, we can mask single channels from Z-stacks within dendrites exclusively, and use object-based co-localization methodology using the “spot” function of Imaris to identify endosomes (Fig. 1A).

To address the endosomal identity of dendritic SEs, neurons were grown for 10–15 days *in vitro* (DIV) and subjected to the FLAG-antibody feeding assay, fixed after a 2–6 hour chase, and co-stained for endosomal markers for early endosomes, recycling endosomes, and late endosomes (Fig. 1B). To mark the early endosome, we utilized an EEA1 antibody as we were unable to find a reliable Rab5 antibody for our purposes. To mark the recycling endosome and late endosomal pools we used Rab11 and Rab7 antibodies respectively. Dendritic SEs showed co-localization with multiple endosomal subtypes, including early endosomes (EEA1), recycling endosomes (Rab11), and late endosomes (Rab7) (Fig. 1C–E). The extent of co-localization was quantified as described in Materials and Methods (Fig. 1A). Analysis revealed that dendritic SEs are overwhelmingly found in two distinct endosomal subtypes, namely Rab11-containing recycling endosomes (32% ± 8) and Rab7-containing late endosomes (41% ± 8) (Fig. 1F), with a roughly even distribution between the two pools. No differences between 2–6 hours were observed, and therefore results were not reported separately. There are two possible pathways for how dendritic signaling endosomes are diversified into Rab11- or Rab7-positive endosomal pools: FLAG-TrkA might already be transported in multiple compartments in the axon to arrive in the soma diversified, or FLAG-TrkA is sorted within the somatodendritic region into two distinct endosomal populations after undergoing retrograde transcytosis. These two pathways are not mutually exclusive and could both occur sequentially.

### Retrograde SE’s are diversified before reaching the cell body

**Figure 2:**
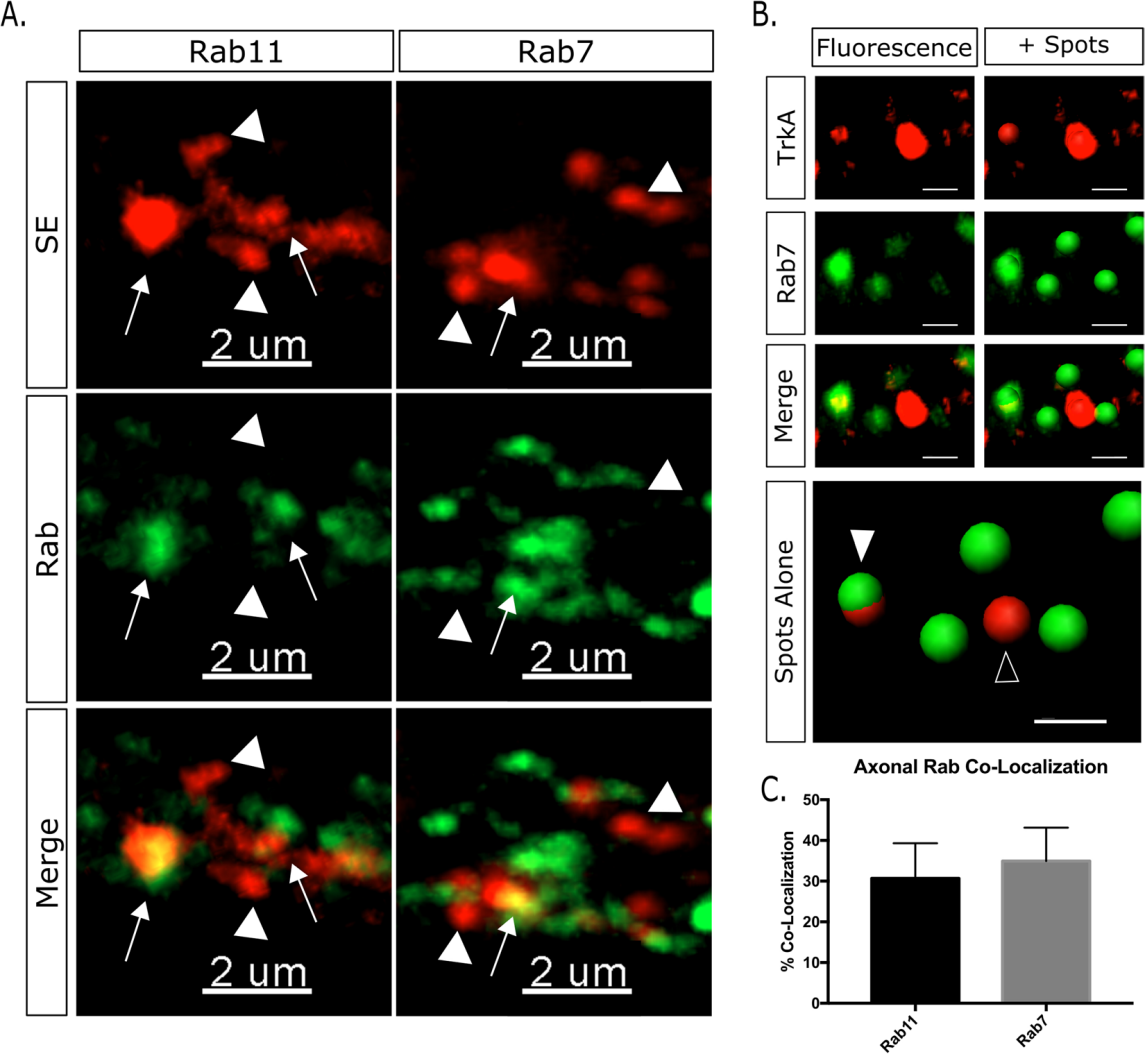
Axonal SEs transport retrogradely in late endosomes and recycling endosomes. (**A**) Retrograde SEs show co-localization with late endosomes (Rab7) and recycling endosomes (Rab11). Arrows are co-localized, arrowheads are not. (**B**) Imaris spot co-localization was used to determine co-localization. (**C**) Quantification of axonal SE co-localization with endosomal marker (n = 3–4 independent experiments, 15 fields of view per experiment).

Our finding that dendritic SEs exist in multiple endosomal populations prompts the question of whether they arrive in multiple distinct compartments, or if SEs diversify upon reaching the dendrite. It is now well established that SEs can travel in the axon in Rab7-positive compartments^28,29^. Late endosomes (Rab7) and, to a lesser extent, recycling endosomes (Rab11), are considered to be retrograde carriers for SEs in axons^29^. To investigate whether Rab7 and/or Rab11 were associated with retrogradely transported signaling endosomes, neurons were subjected to the antibody feeding assay and fixed after 30 minutes when the vast majority of endosomal SE’s are traveling retrogradely^11^. Staining for Rab7, a marker of late endosomes, showed significant co-localization (50.14% +/− 5.28) (Fig. 2A–C). However, approximately half of the signaling endosomes in the axon remained unlabeled (Fig. 2A–C, Rab7). Interestingly, co-staining with Rab11 also showed high co-localization between axonal signaling endosomes and recycling endosomes (46.27% +/− 7.77) (Fig. 2A–C). Axonal signaling endosomes thus show ~50% co-localization with both Rab7 and Rab11, suggesting that two molecularly distinct pools of endosomes are the predominant retrograde carriers for activated TrkA (Fig. 2C). We were unable to find a reliable early endosome antibody for axons, but only a small subset of axonal SEs has been reported to co-localize with Rab5^29^. Therefore, in our hands, retrograde SEs are not molecularly uniform, but are diversified prior to arriving in the soma into recycling and late endosomal pools.

### SEs undergo dynamic endosomal processes in the dendrite

**Figure 3:**
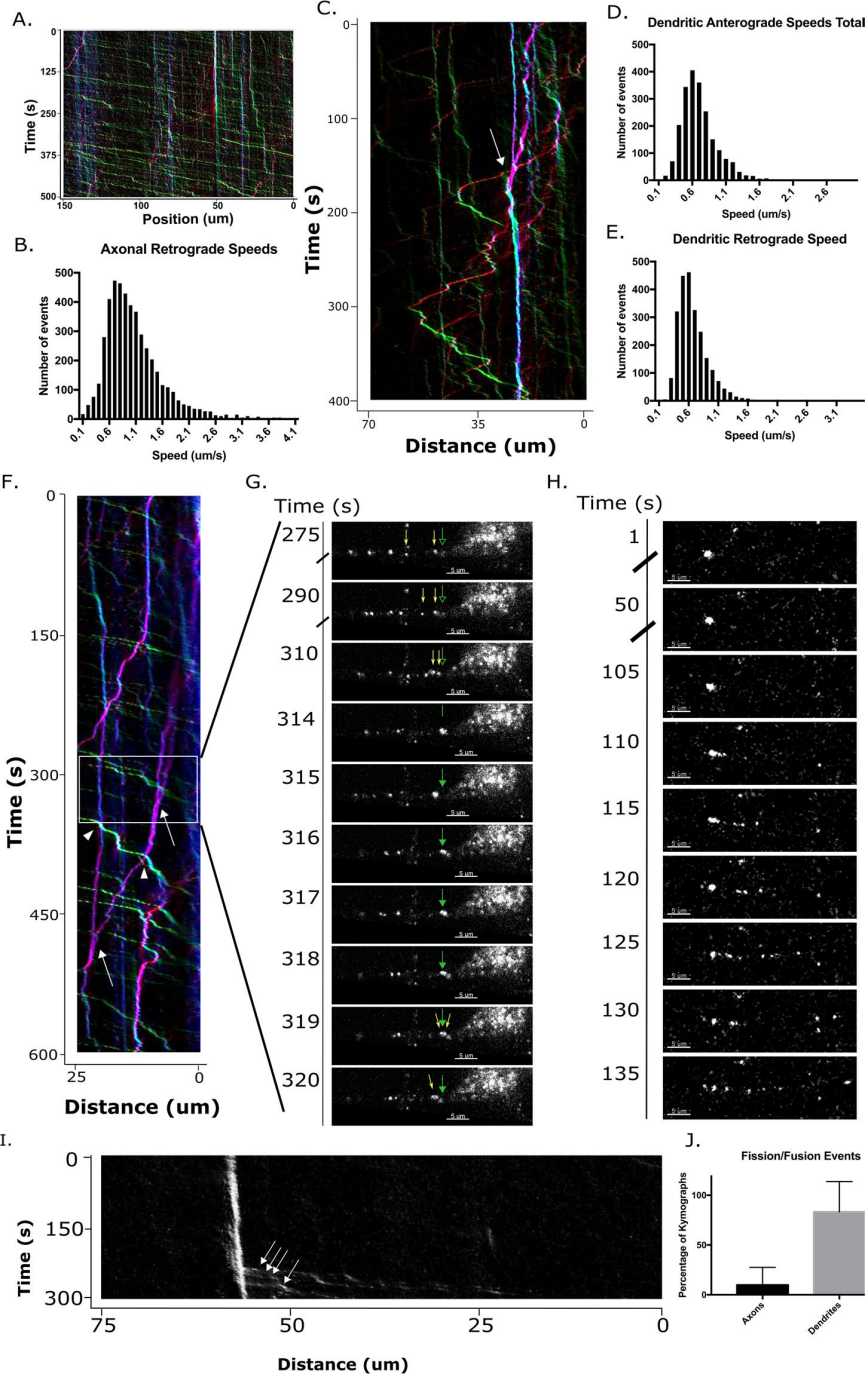
SE movement in dendrites. (**A**) Representative kymograph of retrograde axonal movement of signaling endosomes (visualized with Cy3-anti-FLAG antibody fed in distal chamber). Images were taken at a rate of 1 frame/sec. Kymographs were generated by KymoClear in which stationary events are coded in blue, retrograde events in green, and anterograde events in red. (**B**) Axonal retrograde speeds were calculated from 34 of movies from 3 independent experiments and show an average speed of 0.99 µm/s. (**C**) Representative kymograph of SE movements in a dendrite. Arrow indicates multiple endosomes coming together in a putative fusion event. (**D**) Histogram of anterograde dendritic speeds. Speeds were calculated from 3 independent experiments and show an average speed of 0.71 ± 0.28 µm/s. (**E**) Histogram of retrograde dendritic speeds. Speeds were calculated from 3 independent experiments and show an average speed of 0.67 ± 0.26 µm/s. (**F**) Representative kymograph showing multiple putative fusion events in a single dendrite. Arrows indicate putative fusion between SEs, arrowheads point to an SE that passes with no fusion. Box indicates position of still images in (**G**). (**G**) Stills from dendritic movie showing putative fusion event. Green arrows point at position of putative fusion, and yellow arrows indicate individual endosomes dynamically merging and splitting. (**H**,**I**) A stationary SE undergoing multiple fission events is shown as stills from a dendritic movie (**H**) and as a kymograph (**I**). (**J**) Quantification of putative fission/fusion events in axons vs dendrites. n = 15 cells for axons; 13 cells for dendrites; 3 independent experiments each.

It has been previously reported that SEs in dendrites have distinct movements from axonal SEs^11^, however we find that axonal and dendritic SE pools exist in similar endosomal subtypes. To investigate the different movement profiles between axonal and dendritic SEs, we used a live imaging chamber and a fluorescent anti-FLAG antibody (M2-Cy3, Sigma) and determined the motility of endogenous retrograde SEs in both dendrites and axons. The labeled endosomes correspond to FLAG-TrkA SEs since only background labeling was observed when a control culture not expressing FLAG-TrkA (WT) was incubated with M2-Cy3 antibody. This assay is thus specific for *TrkA*^*FLAG/FLAG*^ neurons (Supplementary Figure 2A), and importantly allows us to visualize endogenous protein trafficking of individual endosomes (Supp. Figure 2B, Movie 1). Similar to previous reports, we find that SEs in the dendrite take frequent pauses, change direction, and move at a slightly lower speed on average compared to axonal SEs (Fig. 3A–E, Movie 2). Interestingly, we noticed incidences where multiple endosomes seemed to merge together into a stationary SE (Fig. 3C, arrow). Dendritic endosomes carrying other cargos (such as transferrin) are capable of fusion and fission, leading to mixing (by fusion) or sorting (by fission) of cargos, but these types of dynamic events have not been reported for the SE. We thus asked if SEs in the dendrite might also undergo dynamic endosomal fission and fusion events. Movies acquired in dendrites were analyzed for putative fusion and fission events. Putative fusion and fission events were scored by the same criteria we have used previously^24^: putative fusions consist of a motile endosome merging with another punctum and not re-emerging on the other side (Movie 3). Putative fission events consist of a single punctum separating into more than one, which usually move away from each other (Movie 4). We refer to these events as “putative” since light microscopy cannot resolve very close apposition of two endosomes from fusion into a single endosome. We find that putative fusion events between SEs in dendrites are observed frequently. Most dendrites (83.3% ± 30.5) displayed at least one putative fusion event and oftentimes more than one within the 7–8 minute movie. A representative kymograph shows two putative fusion events in a single dendrite (Fig. 3F, arrows), however most SEs pass each other unimpeded (Fig. 3F, arrowheads). This indicates that SEs are not a steric hindrance for each other, but that many SEs pass each other without stopping. One representative putative fusion event, corresponding to the arrow boxed in Fig. 3F, is shown in single frames as Fig. 3G.

In addition to putative fusion events, we were able to observe putative fission events where a single large endosome sits stationary for an extended period of time, but then suddenly generates multiple small motile vesicles (Movie 4) (Fig. 3H). Representative kymograph shows small endosomes budding out of a large stationary carrier (Fig. 3I). Intriguingly, these dynamic events suggest that there are multiple pools of signaling endosomes within the dendrite which have the capacity to attach to different motors, tether and fuse with different compartments, and bud out new single endosomes containing axonally-derived TrkA. These dynamic fission and fusion events were almost exclusively observed in the dendrites, and were rare in axons (Fig. 3J).

### Post-Transcytotic Signaling Endosomes (PTSEs) are abundant in dendrites

**Figure 4:**
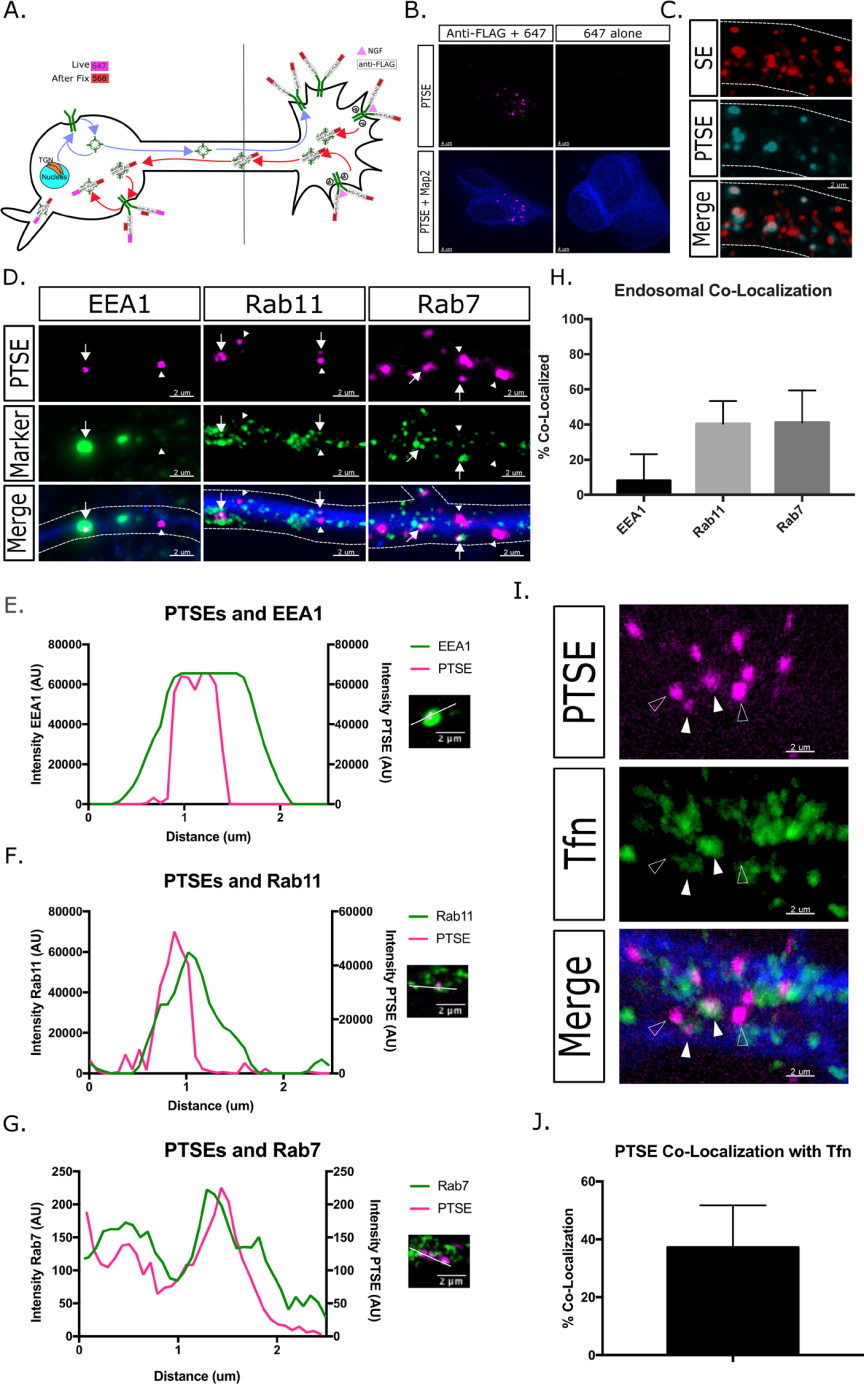
Post-transcytotic signaling endosomes (PTSEs) are abundant in dendrites. (**A**) Antibody feeding assay to label PTSEs as detailed in Materials and Methods. (**B**) PTSE labelling assay is specific for anti-FLAG antibody. No label is detected with the 647 secondary antibody without prior feeding of the anti-FLAG antibody (647 alone). (**C**) Many SEs are labelled by the 647 anti-mouse antibody, indicating that they have undergone retrograde transcytosis. PTSEs are thus abundant in dendrites. Dendrites are outlined with dotted lines. (**D**) After PTSE labelling, PTSEs co-localize with EEA1, Rab11, and Rab7 in dendrites (MAP2 indicated in blue). Arrows indicate co-localized SEs, arrowheads indicate non co-localized SEs. Dendrites are outlined with dotted lines. (**E**–**G**) Representative line scans of intensities showing intensity peaks of EEA1 (**E**), Rab11 (**F**), and Rab7 (**G**) in green and PTSEs in pink. (**H**) Quantification of co-localization of PTSEs with endosomal markers (n = 3–4 independent experiments, 15 fields of view per experiment). (**I**) After 2 hours of dual PTSE and fluorescent Tfn feeding, Tfn (green) substantially colocalizes with PTSEs (purple) in dendrite (MAP2 in blue) (filled arrowheads). Non co-localizing endosomes (empty arrowheads) are also observed. (**J**) Quantification of co-localization between PTSEs and Transferrin (n = 10 fields of view).

The observation that dendritic SEs co-localized to some degree (albeit low) with the early endosomal marker EEA1 in dendrites (7% ± 3) (Fig. 1B–C) suggested that FLAG-TrkA-positive dendritic SEs underwent apparent “maturational reversion” back to an early endosome. We find that SEs are transported down the axon partially as Rab7 positive late endosomes (Fig. 2), consistent with previous studies^17,29–31^. However late endosomes are typically thought of as being on the path towards degradation, not towards reverting back to an early endosome. We additionally find that there is a large population of Rab11 positive recycling endosomes carrying retrograde TrkA in axons. However, recycling endosomes eventually fuse with the plasma membrane, as opposed to reverting back to early endosomes. One possible mechanism of such apparent “maturational reversion” is that dendritic SEs are derived from retrograde transcytosis. As mentioned previously, retrograde transcytosis is a pathway whereby axonally-derived TrkA is exocytosed onto the somatodendritic membrane and subsequently re-endocytosed^7^. We refer to these re-endocytosed SEs as PTSEs (post-transcytotic SEs). The endosomal identities and dynamic behaviors of PTSEs are unknown. To examine if dendritic SEs are PTSEs, we utilized a recycling assay (see Materials and Methods) that specifically marks SEs that have undergone retrograde transcytosis (Fig. 4A). Specifically, we used the distal anti-FLAG antibody feeding assay and, during the chase period, an Alexa 647 anti-mouse secondary antibody was added to the soma chamber. This antibody is capable of binding to the anti-FLAG antibody when it is externalized on the somatodendritic surface and is thus used as a way to track re-endocytosing TrkA which originated at the distal axon. Any naïve (biosynthetic) TrkA pool on the somatodendritic surface was never exposed to the anti-FLAG antibody and therefore would not be bound by the anti-mouse antibody, allowing specific tracking of PTSEs. There is no detectable signal of the Alexa 647-antibody in the *TrkA*^*FLAG/FLAG*^ mouse unless anti-FLAG antibody is added to the distal axon chamber, showing specificity of the labeling procedure (Fig. 4B). Using the recycling assay, we observed that PTSEs are abundant in dendrites (Fig. 4C). Interestingly, PTSEs were rarely observed in axons.

To determine which endosomal subtype PTSEs correspond to, we co-stained for markers against the 3 major endosomal compartments of interest and found co-localization with each compartment (Fig. 4D–G). Similar to total SEs examined above (Fig. 1), PTSEs have low but consistent co-localization with EEA1 (7% ± 4), suggesting that they pass quickly through the early endosome after retrograde transcytosis. They have much higher co-localization with Rab7 (42% ± 9) and Rab11 (36% ± 8), suggesting that they are sorted into both the recycling and degradative arms of the endosomal pathway (Fig. 4H). This suggests that PTSEs exist in multiple endosomal pools within dendrites. Simultaneous detection of FLAG-SEs and PTSEs in dendrites was carried out to determine to what extent dendritic SEs derive from retrograde transcytosis. The proportion was variable but often exceeded 50%. The highest observed proportions exceeded 80%, suggesting that a significant proportion of dendritic SEs undergo retrograde transcytosis. The high variability between different cells was likely due to technical limitations of detecting re-endocytosed pools above background on fainter labeled cells. Alternatively, it is possible that the extent of retrograde transcytosis is heterogeneous among different cells.

### PTSEs mix in with other endosomal cargo

We observed high proportions of PTSEs to be recycling endosomes (Rab11) (Fig. 4D). In addition, we saw frequent dynamic fusion events of SEs in dendrites (Fig. 3J). These observations raised the possibility that re-endocytosing axonally-derived TrkA in dendrites does not stay segregated into a dedicated SE, but dynamically mixes with other cargos entering endosomes from the dendritic surface. We thus sought to determine whether re-endocytosed TrkA (PTSEs) co-localized with other cargos that traverse the dendritic recycling endosome. To this end, we utilized the canonical recycling cargo Transferrin (Tfn). After endocytosis, Tfn is quickly recycled back to the surface of the cell via the early endosome and the recycling endosome. To investigate if re-endocytosed TrkA intermixed with Tfn or stayed separate, we performed an antibody feeding assay where we pulsed NGF and anti-FLAG antibody for 30 minutes on the distal axon side, acid washed to remove remaining surface bound antibody, and incubated the cell body chamber with fluorescent Tfn for 2 hours (Fig. 4I). Such a long incubation with Tfn will result in the filling of both early and recycling endosomes. Interestingly, we see approximately 37% ± 14 of PTSEs positive for Tfn (Fig. 4J). This matches closely with the percentage of PTSEs found in recycling endosomes (Fig. 4H), suggesting that the re-endocytosed axonally-derived TrkA present in recycling endosomes mixes in with other cargos fated for the same destination.

**Figure 5:**
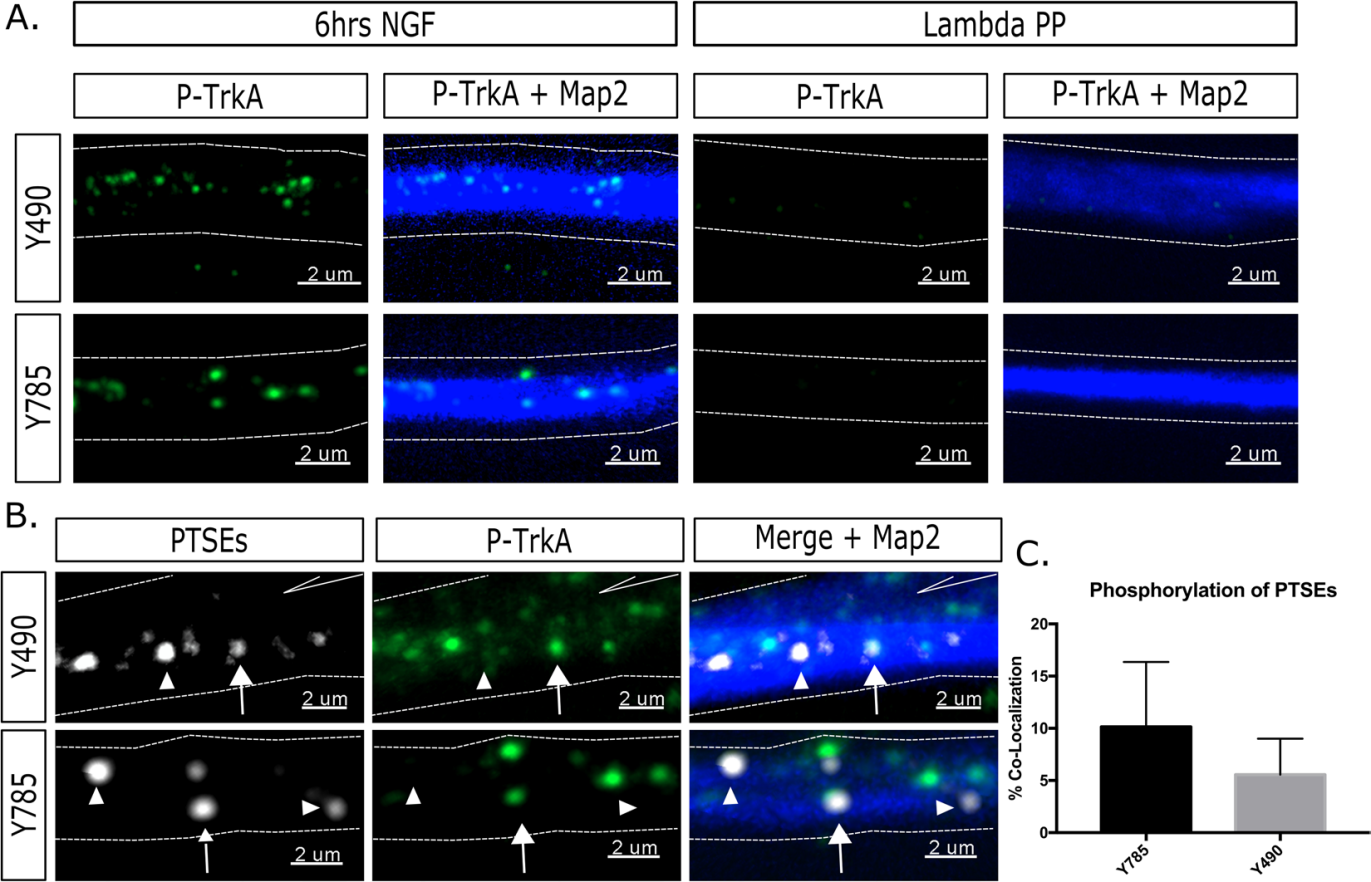
A subset of PTSEs contain activated TrkA. (**A**) Antibodies against phosphorylated TrkA (p-Y490 and p-Y785) show high signal in dendrites (as shown by MAP2) after 6 hours of NGF feeding, but signal is abolished when neurons are treated post-fixation with Lambda Phosphatase. Dendrites are outlined with dotted lines. (**B**) PTSEs partially co-localize with p-Y490 and p-Y785. Arrows are co-localized, arrowheads are not co-localized. Dendrites are outlined with dotted lines. (**C**) Quantification of co-localization between PTSEs and P-TrkA (n = 2–3 independent experiments, 15 fields of view per experiment).

In order to determine if TrkA was still signaling after retrograde transcytosis, we counterstained against two phosphorylation sites on TrkA which are associated with active signaling of TrkA, pY490 and pY785. Specificity of antibodies was tested by treating the cells with Lambda Phosphatase prior to immunostaining. We observed loss of both phosphorylated antibody signals after phospatase treatment (Fig. 5A). Previous work reported that between 15 and 35% of dendritic SEs still had associated phosphostaining^11^. Since a high proportion of dendritic SEs can arise via retrograde transcytosis, we expected to see co-labeling of PTSEs with anti pTrkA antibodies in a similar range. Indeed, some PTSEs are marked with antibodies against p-Y490 (5.56 ± 0.8%) or p-Y785 (10.13 ± 0.9%) (Fig. 5C), suggesting that at least a subset of PTSEs are still signaling.

### PTSEs have distinct movement profiles

**Figure 6:**
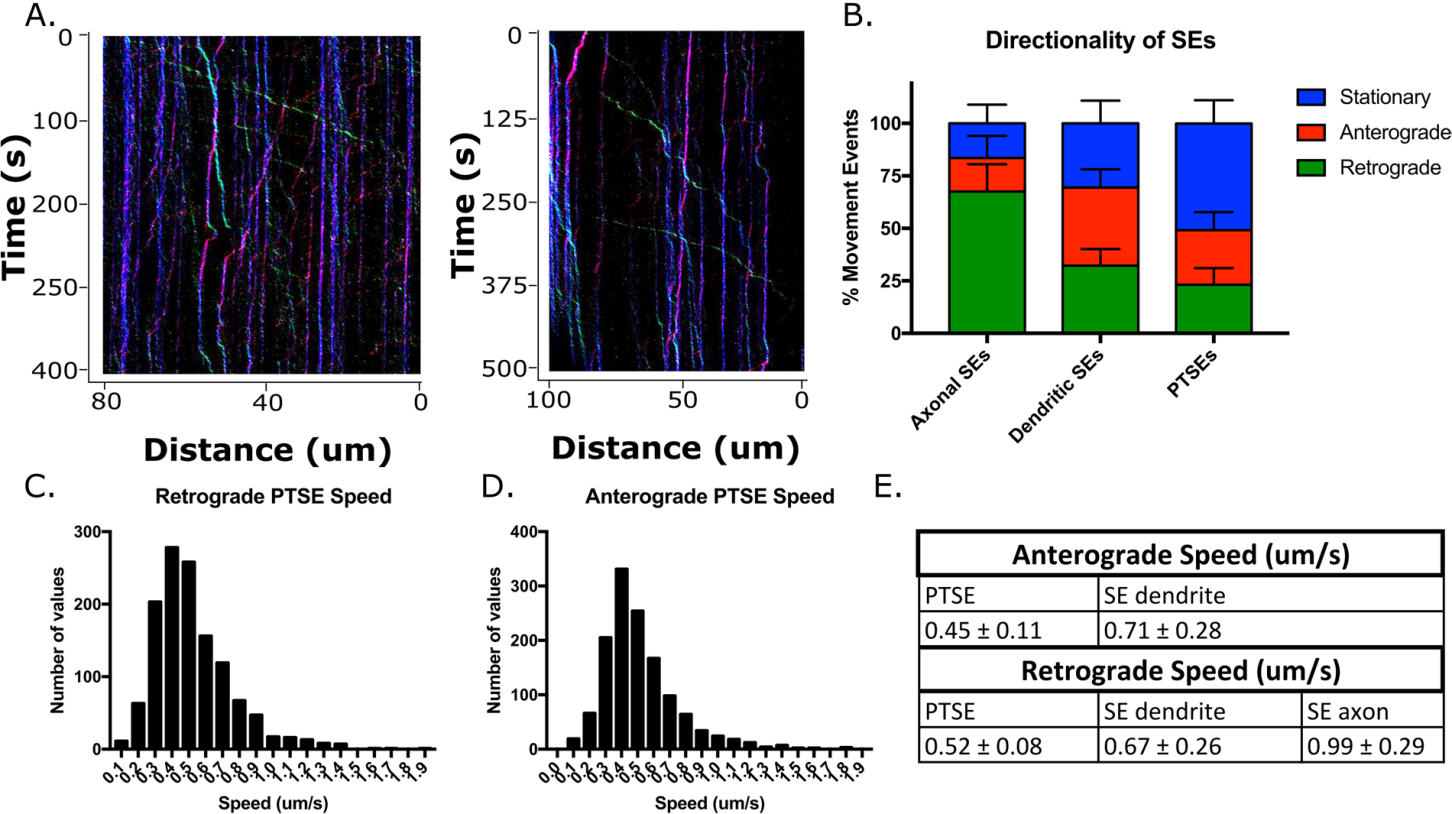
PTSE movement in dendrites. (**A**) Representative kymographs showing many stationary endosomes (blue) and long-range movements (red = anterograde and green = retrograde). (**B**) Quantification of directionality (n = 15–30 kymographs from 4 independent experiments). Each direction for the three population were significantly different from each other (i.e. percent anterograde was significant between axons and dendrites, axons and PTSEs, and dendrites and PTSEs) at p < 0.0001, except retrograde movement between dendritic SEs and PTSEs, which was significant at p = 0.0014 as determined by ANOVA. (**C**) Dendritic anterograde PTSE speeds were calculated from 24 movies from 4 independent experiments. (**D**) Dendritic retrograde PTSE speeds were calculated from 24 movies from 4 independent experiments. (**E**) Quantification of PTSE speeds using KymoClear and KymoDirect comparing axonal SEs, dendritic SEs and dendritic PTSEs.

We next determined the movement profiles of PTSEs. To this end, we utilized the same PTSE assay as for fixed cells (Fig. 4A) and carried out live imaging with fluorescent secondary antibodies to capture re-endocytosing FLAG-antibody decorated TrkA in the somatodendritic domain. We observed many examples of fast persistent movements similar to that of dendritic SEs, showing that PTSEs are capable of long-range movement (Fig. 6A). Interestingly, there is a large pool of stationary endosomes, pseudocolored as blue on kymographs (Fig. 6A). The percentage of stationary endosomes is much larger for PTSEs than it is for axonal or dendritic SEs (Fig. 6B). These differences are statistically significant. Additionally, the instantaneous speeds of PTSEs are slightly slower compared to SEs in the dendrites and axons, both for the anterograde and retrograde direction (Fig. 6C–E). It is possible that stationary PTSEs play a distinct signaling role within the dendrites, however we cannot rule out that the large stationary pool arises from technical differences in the two assays for PTSE and SE live imaging rather than biological differences. Visualization of PTSEs requires application of a secondary labeled antibody to the live cells. This extra antibody layer could cause less efficient internalization or reduced motility.

### SEs are diversified *in vivo*

**Figure 7:**
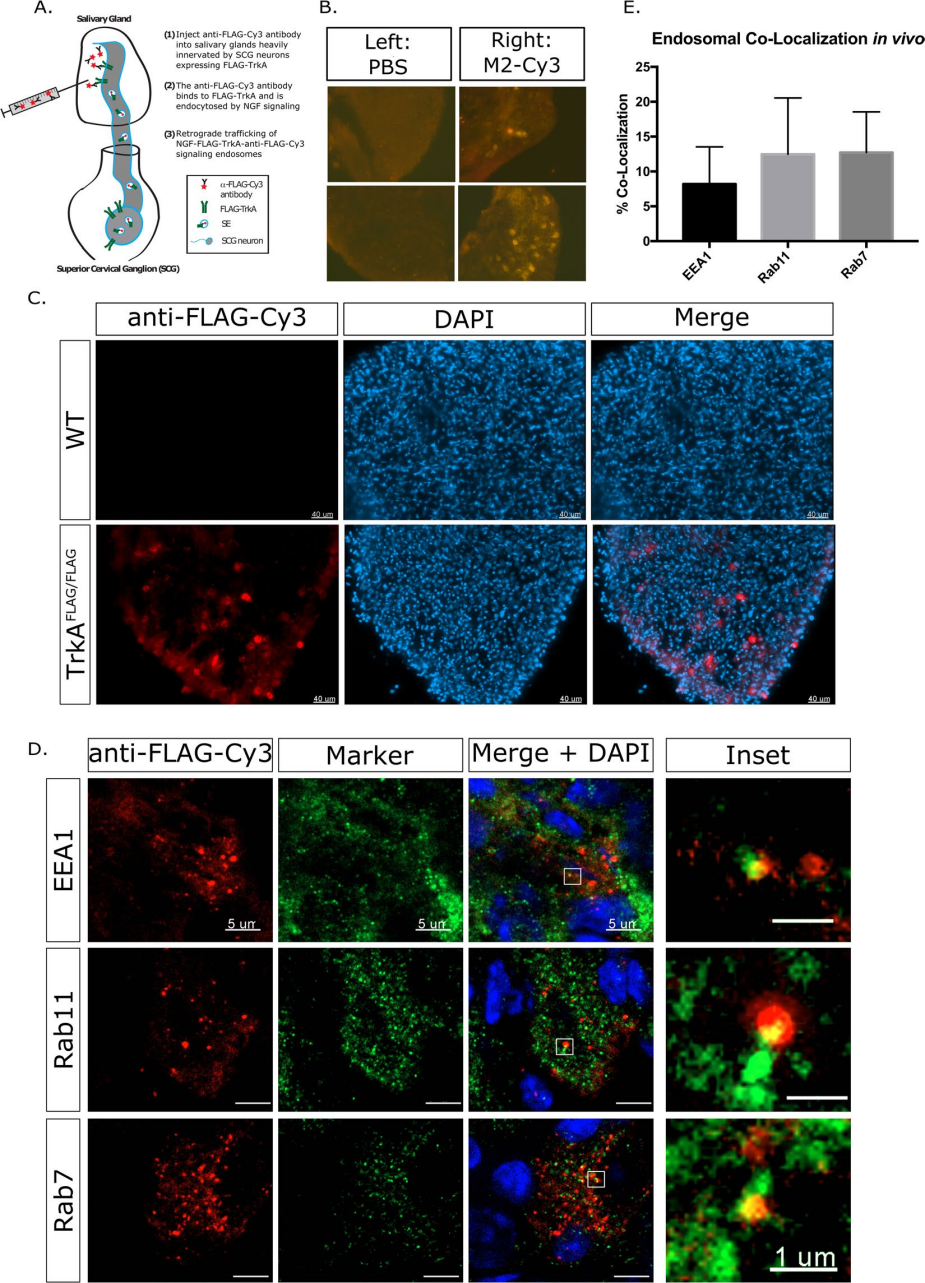
SEs are diversified *in vivo*. (**A**) SEs can be labelled *in vivo* by injection into the salivary glands, shown diagrammatically. (**B**) Specificity of SE labelling. Injection of M2-Cy3 into the right eye and PBS into the left eye shows exclusive signal in the ipsilateral (right) SCG but not the contralateral (left) SCG. (**C**) Bright antibody labelling is detected when M2-Cy3 is injected into the salivary gland of *TrkA*^*FLAG/FLAG*^ animals, but no label is observed when M2-Cy3 is injected into WT (non-FLAG) animals. (**D**) Close up imaging of single cells shows partial co-localization of *in vivo* SEs in the SCG with EEA1, Rab11, and Rab7. Scale bar 5 µm, inset scale bar 1 µm. (**E**) Quantification of SE co-localization with Rab proteins (n = 30–40 cells from 2 independent experiments).

Next, we asked whether or not SEs are diversified into Rab11 recycling endosomes and Rab7 late endosomes *in vivo* as they are *in vitro*. A previously published paper^11^ labeled endosomes *in vivo* by injection of labeled WGA into the anterior eye, a target region for a subset of SCG axons. They show that WGA-containing endosomes travel retrogradely and label cell bodies within the SCG. Wheat germ agglutin is a lectin that binds to carbohydrate chains on many membrane proteins, and therefore the exact relationship between WGA-containing endosomes and TrkA-SEs is not known. In order to unambiguously label FLAG-TrkA SEs, we injected a fluorescent anti-FLAG antibody into the eye or the salivary gland, which is also innervated by the SCG (Fig. 7A). We observed clear FLAG-antibody decorated SEs in the SCG of eye-injected FLAG-TrkA mice, but not in FLAG-TrkA mice eye-injected with PBS, showing that we are able to visualize TrkA-SEs *in vivo* (Fig. 7B), similarly to what was previously shown^11^. Additionally, injection into the eye labelled projections exclusively to the ipsilateral SCG, not the contralateral SCG, showing that the antibody is being contained within the eye and SCG and not spreading to the contralateral side of the body (Fig. 7B). No label was observed when anti-FLAG antibody was injected into salivary glands of WT mice (another target of SCG axons) not expressing FLAG-TrkA, showing assay specificity (Fig. 7C).

To ask if SEs *in vivo* are diversified, *TrkA*^*FLAG/FLAG*^ animals received anti-FLAG antibody injections in the salivary gland, and their somata in the SCG were subsequently examined for Rab co-localization. Numerous labeled cells that successfully took up the antibody are clearly visible within the SCG (Fig. 7C). Fixed sections were counter-stained with antibodies towards endosomal markers including early endosomes (EEA1), recycling endosomes (Rab11), and late endosomes (Rab7). Retrograde SEs partially co-localize with each of these markers *in vivo*, suggesting that retrograde SEs are diversified into recycling and late endosomal pools in the soma *in vivo* as well (Fig. 7D,E).

## Discussion

NGF was the first neurotrophic factor discovered, and it has served as the archetypal ligand-receptor system in the nervous system to study the molecular underpinnings of nervous system development, maintenance, and pathology. How precisely the signal from target-derived NGF is transduced from distal tips of axons long distances back to the cell body has been of intense interest for over 20 years and has led to the important discovery that signaling takes place on endosomes after endocytosis of NGF-TrkA. The term “signaling endosome” was coined specifically based on the findings from the NGF-TrkA system^32^. It is now well established that the NGF-TrkA signaling endosome travels retrogradely from the axon terminal to the soma to mediate a multitude of developmental events in time and space^33^. We also know that retrograde SEs arriving at the soma from the axon can undergo additional trafficking events, including entering dendrites and undergoing rounds of exo- and endocytosis in the soma (termed retrograde transcytosis)^7^. Very little is known, though, about these somatic and dendritic SE trafficking events. We show here that dendritic SEs undergo dynamic fusion and fission events, mix with other endocytosed cargos in recycling endosomes, and do not stay as a distinct endosomal entity in dendrites. In contrast, axonal SEs undergo few dynamic fusion events during their retrograde transport even though they exist in the same endosomal sub-populations (Fig. 3J). We thus propose a model whereby TrkA travels in the axon in distinct, non-intermixing SEs, but then undergoes additional molecular diversification into recycling and late endosomes by undergoing retrograde transcytosis in the somatodendritic domain. Dendritic SEs thus undergo dynamic trafficking events and intermix extensively with other endocytosing cargos.

After NGF-TrkA binding and internalization in the distal axon, we and others have determined that ~40–50% of retrograde SEs are Rab7-containing late endosomes^17^ (Fig. 2). Rab7-containing late endosomes are typically thought of as a pre-degradative compartment^34^. Additionally, late endosomes are typically multivesicular bodies (MVBs) with intraluminal vesicles (ILVs). It has been previously suggested that there are multiple morphologically distinct pools of SEs in the axon, including MVBs and single vesicles^30^. How neurotrophic receptors residing on ILVs can induce critical neurotrophic functions such as survival or PSD clustering remains unknown. Here, we report the first observed fusion and fission events of SEs (Fig. 3), which is consistent with packing or unpacking SEs to and from MVBs into additional endosomal pools. Dynamic fusion and fission events are commonly observed in other cell types and with other cargos within the endosomal system. Fission and fusion are indicative of sorting events taking place. We propose a new model based on our findings that NGF-TrkA is able to dynamically sort away from degradation and recycle instead.

We previously identified NGF-TrkA in recycling endosomes in the soma, however the mechanism by which it arrived in the recycling endosome (Rab11) was not established^7^. This created a conceptual challenge because late endosomes (Rab7) were considered the sole carrier of retrograde TrkA. Here we reconcile this by finding that that the SE travels retrogradely in roughly equal proportions of Rab7+ and Rab11+ carriers (Fig. 2). Based on our data, we propose a model whereby upon internalization at the distal tips of axons, NGF-TrkA rapidly moves through an early endosome compartment and diversifies into 2 major retrograde carriers: recycling endosomes and late endosomes. While this is the first evidence that a substantial pool of SEs can travel retrogradely in a recycling endosome, others have found that anterogradely transporting TrkA and post-endocytic TrkA in the cell body can co-localize with Rab11^7,14^.

Rab11 endosomes recycle cargo to the surface of the cell, whether in a similar location or somewhere far away. Therefore, it was an intriguing possibility that recycling endosomes may be fated towards retrograde transcytosis. This retrograde transcytosis may then allow re-endocytosed NGF-TrkA to enter Rab11 carriers. We indeed find that re-endocytosed retrograde TrkA sorts quickly out of the early endosome equally into the recycling (Rab11) and degradative (Rab7) arms of the endosomal pathway, providing a novel model of diversification for TrkA signaling endosomes.

What roles do distinct pools of NGF-TrkA endosomes play in the development of a neuron? An intriguing possibility is that the function of the SE is partitioned into molecularly discrete pools. The SE is able to initiate many different signaling cascades, each with distinct functional outcomes. In the axon, SEs are able to signal locally to enhance axon growth and branching^5,35^. Additionally, they can signal through PI3K to promote internalization and retrograde transport^36^. In the soma, they are able to signal through calcium and MAPK to promote CREB phosphorylation and large transcriptional changes^37,38^. How is the signaling endosome able to initiate so many unique signaling cascades? One possibility is that endosomal diversity could lead to functional diversity between endosomes. For example, recruitment of Coronin-1a in the cell body endows the SE with the ability to induce calcium release, avoid lysosomal fusion, and undergo retrograde transcytosis^7^. Interestingly, Coronin1a also plays a role in axon outgrowth by affecting PI3K signaling, but does not affect MAPK signaling^35^. Because of their distinct maturational and trafficking capacities, an intriguing possibility is that these pools have distinct signaling capacities. Indeed, the trafficking and signaling of cargo, including TrkA, are often intertwined^39^. Therefore, the multiple trafficking patterns (late and recycling endosomal carriers) could influence endosomal maturation and signaling function. Much more work will need to be done to elucidate these potential signaling patterns.

Another interesting possibility for the role of multiple endosomal populations within the dendrite is the development of post-synaptic densities. TrkB and BDNF signaling play a critical role in synaptic development in neurons within the central nervous system, specifically with regard to glutamatergic synapses^40–42^. As opposed to NGF and TrkA’s canonical role as retrograde carriers for neurotrophic signaling, it has been shown that BDNF can be released locally and act in a paracrine manner within the dendrites during long-term potentiation^43^. Interestingly, during long term potentiation (LTP), BDNF-dependent TrkB endocytosis results in the recycling of TrkB to the surface via the Rab11 recycling endosome. This recycling via Rab11 is critical for sustained TrkB signaling, but unnecessary for transient signaling^44^. Interestingly, internalized BDNF also gets recycled back to the surface in activity-dependent secretion. This recycled BDNF is then available to act again locally at the dendrite. This recycled pool of BDNF is critical for maintaining LTP^45^. This draws many parallels to the retrograde transcytosis pathway in sympathetic neurons, and begs the question: Is NGF being released by SEs that undergo retrograde transcytosis? Indeed, only a subset of PTSEs retain phosphorylated TrkA, indicating that they may have lost the bound NGF. The fate of NGF, the importance of TrkA recycling back to the surface, and the role retrograde transcytosis plays in the development of dendritic specializations in sympathetic neurons will be an intriguing question for the field.

In addition to retrograde transcytosis, Kuruvilla and co-workers have discovered an additional transcytotic pathway which is distinct from the retrograde transcytosis pathway described by us^14,46^. Their work shows that naïve TrkA travels anterogradely by transcytosis from the soma surface to the axon. Interestingly, this is dependent on signaling from retrogradely arriving NGF-TrkA. In their recent work, they propose an intriguing model by which the NGF-TrkA SE is exocytosed on the soma, trans-phosphorylates and stimulates the endocytosis of unliganded naïve TrkA through interaction of the naive receptor with the axonally-derived TrkA. The naive receptor is then able to undergo endocytosis. After endocytosis, the naïve receptor is de-phosphorylated by PTP-1B and is trafficked anterogradely into the axon^46^. This creates a feedforward loop whereby recycled NGF-TrkA induces anterograde trafficking of newly synthesized naïve TrkA. Intriguingly, this anterogradely transported naïve TrkA pool also travels in Rab11 recycling endosomes^14^. However, it remained unclear as to what happened to the axonally-derived TrkA that interacts with naive TrkA. Our work specifically follows axonally-derived TrkA, and we interestingly do not see PTSEs traveling anterogradely into axons but instead find them throughout dendrites. Even after 6 hours, we see few to no PTSEs in axons. How the naïve TrkA pool which re-endocytoses together with axonally-derived TrkA from the somatodendritic surface sorts away into a distinct axon-bound Rab11 endosome is completely unknown at this point, but will be of great importance to discover in the future.

In summary, our findings provide the first evidence of molecular diversification of NGF-TrkA SEs. We find that there are two mechanisms of diversification: initial diversification of axonal retrogradely transporting SEs, and subsequent diversification through retrograde transcytosis. The SE is additionally able to undergo dynamic fusion events, as well as mixing with other somatodendritically endocytosed cargos, showing that it is much more dynamic than has been previously appreciated. Our results suggest the hypothesis that functional diversification of NGF signaling might be achieved by molecular diversification of SEs, a hypothesis we will test in future experiments.

## Electronic supplementary material

Movie 1

Movie 2

Movie 3

Movie 4

Supplementary Information
