# Supplementary material for: Transcytosis of TrkA leads to diversification of dendritic signaling endosomes
Source: Sci Rep. 2018 Mar 16;8:4715. doi: 10.1038/s41598-018-23036-8 (PMC5856830; doi:10.1038/s41598-018-23036-8)

Transcytosis of TrkA leads to diversification of dendritic signaling endosomes.

Authors:

Kelly Barford[1], Austin Keeler[2], Lloyd McMahon[1], Kate McDaniel[1], Chan Choo Yap[1], Christopher D. Deppmann[2,3,4], Bettina Winckler[1,4].

Movie 1. Axonal signaling endosomes moving retrogradely from the distal axon chamber through the microgrooves towards the cell body chamber.

Movie 2. Movement of dendritic signaling endosomes within the cell body chamber. Dendrites outlined in white.

Movie 3. Putative fusion events in dendrites. Arrow shows three small endosomes coming together and moving together.

Movie 4. Putative fission events in dendrites. Arrow shows large stationary carrier, and smaller single arrow shows one of many carriers budding out from the initial stationary carrier.

Supplementary Figure 1. Microfluidic devices were used to isolate cell bodies (CB) from distal axons (DA). (A) Microfluidic devices. (B) Cell bodies and dendrites, as marked by MAP2, were separated from distal axons, as marked by Tau, by microgrooves. (C) Anti-FLAG antibody application for 30 minutes shows staining exclusively in *TrkA*$^{FLAG/FLAG}$ neurons and not WT neurons. (D) Defining terms. (E) Summary diagram of anterograde transcytosis (blue arrows) versus retrograde transcytosis (black arrows). The two axonal pools of retrograde SEs are indicated by red arrows (Rab7) and green arrows (Rab11)

A. B.

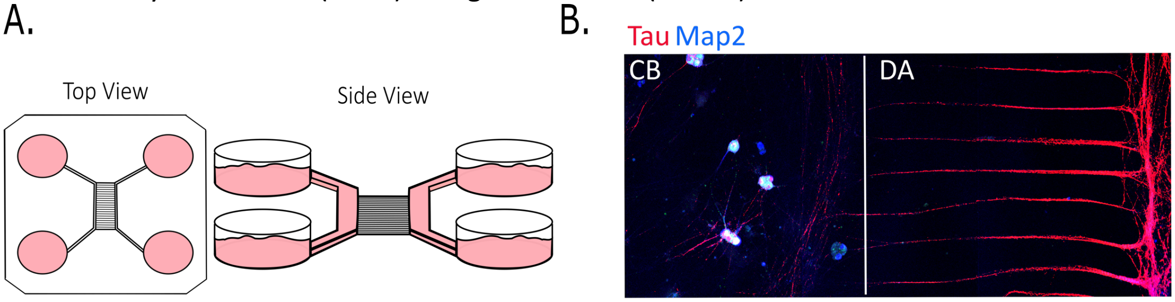

C.

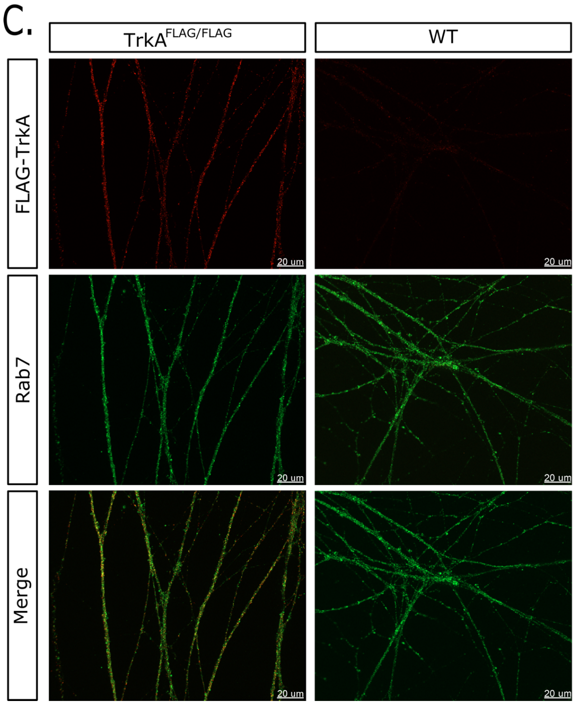

D.

Abbreviation	Definition
SE	Signaling Endosome: Any endosome containing axonally-fed anti-FLAG antibody in presence of NGF
PTSE	Post-Transcytotic Signaling Endosome: Any endosome containing axonally-fed anti-FLAG antibody in presence of NGF, and subsequently bound anti-FLAG secondary antibody on the somatodendritic surface

E.

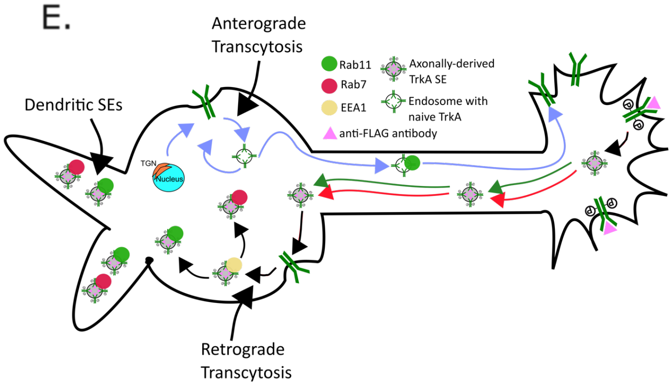

Supplementary Figure 2. Specificity of live imaging paradigm. (A) anti-FLAG antibody feeding assay is specific for *TrkA*$^{FLAG/FLAG}$ mice compared to WT mice. Axons shown with CTXB-488. (B) Individual tracking of endosomes.

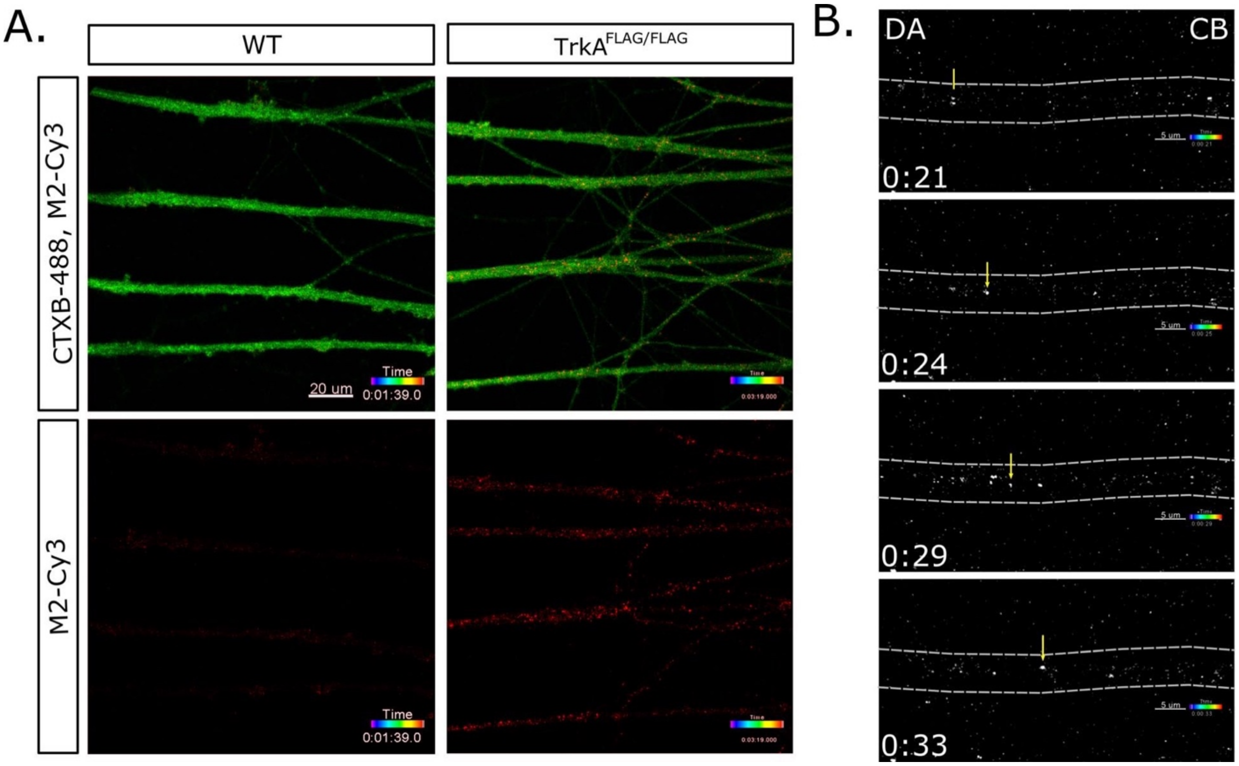

Supplement: Supplementary file 5 — Supplementary Information [file 41598_2018_23036_MOESM5_ESM.pdf]
